# *Bifidobacterium longum* Subspecies *infantis* Bacteremia in 3 Extremely Preterm Infants Receiving Probiotics

**DOI:** 10.3201/eid2209.160033

**Published:** 2016-09

**Authors:** Eirin Esaiassen, Pauline Cavanagh, Erik Hjerde, Gunnar S. Simonsen, Ragnhild Støen, Claus Klingenberg

**Affiliations:** University Hospital of North Norway, Tromsø, Norway (E. Esaiassen, P. Cavanagh, G.S. Simonsen, C. Klingenberg);; Arctic University of Norway, Tromsø (E. Esaiassen, E. Hjerde, G.S. Simonsen, C. Klingenberg);; St. Olavs Hospital, Trondheim, Norway (R. Støen);; Norwegian University of Science and Technology, Trondheim (R. Støen)

**Keywords:** Bifidobacterium longum subspecies infantis, bacteria, bacteremia, preterm infants, probiotics, oral probiotics, Infloran, probiotic supplementation, Norway

**To the Editor:** Metaanalysis of randomized trials that tested different probiotics showed a reduction of ≈50% in necrotizing enterocolitis and all-cause deaths in preterm infants (*1*). Use of probiotics is increasing worldwide (*2*,*3*), and cases of probiotic sepsis were not reported among >5,000 infants in an updated review (*1*). 

In Norway, a consensus-based protocol recommending prophylactic probiotic supplementation for preterm infants at highest risk for necrotizing enterocolitis (gestational age <28 weeks, birthweight <1,000 g) was introduced in 2014. After considering the safety profile, we investigated use in preterm infants of a widely used combination of oral probiotics (Infloran; Laboratorio Farmacéutico **Specialità Igienico Terapeutiche,** Mede, Italy) that contained 10^9^ *Lactobacillus acidophilus* (ATCC 4356) and 10^9^ *Bifidobacterium longum* subspecies *infantis* (ATCC 15697).

*B. longum* is a microaerotolerant, anaerobic bacterium susceptible to many antimicrobial drugs (Table). This bacterium is a rare cause of neonatal infections; until 2015, only 2 *Bifidobacterium* bacteremia cases in premature newborns had been reported (*4*,*5*).

**Table:** Characteristics of 3 extremely preterm infants with *Bifidobacterium longum* subspecies *infantis* bacteremia, 2015*

Characteristic	Patient 1	Patient 2	Patient 3
NICU	A	B	A
Sex	M	M	F
Date of onset	Apr	Jul	Sep
Gestational age, wk	24	23	24
Birth weight, g	730	500	697
Mode of delivery	Vaginal	Vaginal	Caesarean section
Apgar score at 1, 5, and 10 min after birth	4, 5, 5	Unknown, 0, 4	2, 2, 3
Reason for prematurity	Preterm rupture of membranes, maternal infection	Sudden preterm rupture of membranes, delivery not attended by healthcare personnel	Placental abruption
Age at onset of sepsis, d	8	12	46
Maximum CRP level, mg/L, <48 h of symptom onset	147	25	242
Age at discharge, wk	40	41	43
Weight at discharge, kg	3.3	3.4	3.3
Bacterial culture medium and conditions	BacT/ALERT,† aerobic, 36°C	BACTEC Plus,† aerobic, 35°C	BacT/ALERT,† aerobic, 36°C
Bacterial growth in blood culture, d	2	3	2

*Patients were given ½ to 1 capsule/day of oral probiotics (Infloran; Laboratorio Farmacéutico **Specialità Igienico Terapeutiche,** Mede, Italy) that contained 10^9^ *Lactobacillus acidophilus* (ATCC 4356) and 10^9^ *B. longum* subspecies *infantis* (ATCC 15697). MICs (mg/L) for antimicrobial drugs tested were 0.016 for meropenem, 0.032 for ampicillin, 0.064 for penicillin, 0.064 for piperacillin/tazobactam, 0.250 for cefotaxime, 0.250 for clindamycin, 0.250 for vancomycin, and 4.000 for ciprofloxacin. All bacterial strains were inherently resistant to aminoglycosides. ATCC, American Type Culture Collection (Manassas, VA, USA); CRP, C-reactive protein; NICU, neonatal intensive care unit. †bioMérieux (Marcy l’Étoile, France).

A total of 290 extremely preterm infants received oral probiotics during April 2014–August 2015 in Norway. Three patients were given a diagnosis of *B. longum* bacteremia: 2 patients in a neonatal unit in which 17 patients were given oral probiotics and 1 patient in a neonatal unit in which 31 patients were given oral probiotics (Table).

All 3 infants had respiratory distress syndrome and received mechanical ventilation after birth. Enteral feeding with human milk was begun on day 1. Oral probiotics (½ capsule, 1×/d) were given during the first week of life and increased to 1 capsule/day after 4–7 days.

We identified *B. longum* in blood cultures by using matrix-assisted laser desorption/ionization time-of-flight mass spectrometry (Bruker Daltonics, Billerica, MA, USA). Whole-genome sequencing (MiSeq, Illumina, San Diego, CA, USA) and comparative analysis of nucleotide-level variation by using variant cell format in SAMtools (http://samtools.sourceforge.net) showed that all 3 blood culture isolates and a *B. longum* strain cultured from an oral probiotic capsule were identical.

Patient 1 had sepsis and severe hypotension 8 days after birth. A blood culture was prepared, and the patient was given antimicrobial drugs and vasoactive support. Abdominal distention, gastric residuals, and feed intolerance developed the next day, but the patient was cardiorespiratory stable. On day 12, abdominal radiographs showed pneumoperitoneum. Surgery showed multiple ileal perforations and bowel necrosis. Histologic analysis showed classical features of necrotizing enterocolitis. The patient received an ileostoma and improved after treatment with antimicrobial drugs. Blood culture was positive for gram-positive rods, which were identified as *B. longum*. Subsequent clinical course was uneventful.

Patient 2 had apnea, bradycardia, and temperature instability 12 days after birth. A blood culture was prepared, and the patient was given antimicrobial drugs. Blood culture was positive for gram-positive rods, which were identified as *B. longum*. Use of oral probiotics was discontinued. The patient recovered rapidly, and subsequent clinical course was uneventful.

Patient 3 had sepsis and necrotizing enterocolitis 9 days after birth. Ultrasound showed free abdominal fluid. A blood culture was prepared, and the patient was given antimicrobial drugs. Surgery showed 2 separate bowel perforations, and the patient received an ileostoma and colostoma. Histologic analysis did not show necrosis or inflammation. *Enterococcus faecalis* grew in the blood culture obtained on day 9. The patient had a complicated clinical course and received prolonged mechanical ventilation. However, the patient gradually tolerated full feeds. Use of oral probiotics was continued.

On day 46, the condition of patient 3 suddenly deteriorated; hypotension and metabolic acidosis developed, and the patient was again given antimicrobial drugs. A blood culture was positive for *B. longum*. Supplementation with oral probiotics was discontinued. The patient recovered from the infection, but secondary ileus developed. The patient had a complicated clinical course until discharge.

Recently, 5 other *B. longum* bacteremia cases among 5 preterm infants at 26–31 weeks gestation were reported (*6*,*7*). All 5 infants had received oral probiotics; 3 had severe gastrointestinal complications, similar to patient 1 in our report, and 2 patients were moderately compromised, similar to patient 2 (*6*,*7*).

We do not know whether *Bifidobacterium* organisms in blood culture for patient 1 were a consequence of intestinal necrosis and bacterial translocation or the cause of necrotizing enterocolitis. Patient 3 probably had a leaky gut that predisposed this patient to bacterial translocation. All 3 patients were extremely premature (23–24 weeks gestation) and had impaired immune systems, which predisposed them to infections with bacteria with low virulence. A recently published case of *Bifidobacterium* bacteremia in a 2-year old boy with leukemia highlights impaired immunity as a risk factor (*8*).

Only aerobic blood cultures are prepared for neonates. We detected *Bifidobacterium* bacteremia by using 2 automated blood culture systems and aerobic bottles. However, the sensitivity of these systems for detecting *Bifidobacterium* bacteremia is unknown. Thus, the incidence of *Bifidobacterium* bacteremia is theoretically underestimated. Matrix-assisted laser desorption/ionization time-of-flight mass spectrometry improves species detection and its use might be 1 reason for the apparently recent increase in probiotic-associated bacteremia.

We report that systemic infection with probiotic bacteremia might have a severe clinical course in extremely preterm infants. Clinical suspicion and appropriate blood culture conditions are essential for proper diagnosis and management.
